# Cloning and functional analysis of the lobed-leaf gene *BjA10.LL* in *Brassica juncea* L.

**DOI:** 10.1007/s44154-025-00280-3

**Published:** 2026-01-20

**Authors:** Jiajia Liu, Yunyun Ma, Yuxuan He, Xiaohui Cui, Shaojie Ma, Zijin Liu, Saiqi Yang, Yuan Guo, Mingxun Chen

**Affiliations:** State Key Laboratory for Crop Stress Resistance and High-Efficiency Production, and College of Agronomy, Northwest A&F University, Yangling, Shaanxi 712100 China

**Keywords:** *Brassica juncea* L., Leaf margin lobation, Gene mapping, HD-ZIP I protein, Cis-regulation

## Abstract

Leaf shape plays a crucial role in plant growth and development. Among various leaf traits, marginal lobation serves as an ideal morphological marker for breeding programs. However, the genetic mechanism underlying leaf margin lobation in *Brassica juncea* L. remains unclear. Through RNA sequencing and map-based cloning, we identified an incompletely dominant gene, *BjA10.LL*, which encodes an HD-ZIP I protein and is responsible for the formation of leaf margin lobation in *B. juncea*. Sequence analysis of parental alleles revealed no critical variations in the coding region but identified substantial variations in regulatory regions. Heterologous expression of *BjA10.LL* in *Arabidopsis thaliana* confirmed its sufficiency to induce lobed leaves. To functionally link the regulatory variations to the phenotype, we analyzed promoter activity and developed a co-dominant molecular marker targeting key indels in a core enhancer. The promoter activity was significantly affected by these sequence variations, and the marker exhibited perfect co-segregation with the lobed-leaf phenotype in an F₂ population, collectively establishing these regulatory polymorphisms as the causal basis for divergent *BjA10.LL* expression and leaf morphology. These results demonstrate that *BjA10.LL* positively regulates marginal lobe formation, providing insights into leaf shape regulation in *B. juncea* and facilitating the genetic improvement of rapeseed.

**Supplementary Information:**

The online version contains supplementary material available at 10.1007/s44154-025-00280-3.

## Highlights

The study developed seven SSR and two IP molecular markers linked to the leaf margin lobation gene *BjA10.LL* in *B. juncea*.

The study revealed that cis-regulatory variations of *BjA10.LL* gene play a crucial role in leaf margin lobation in *B. juncea*.

The study demonstrated that a co-dominant molecular marker targeting indels in the *BjA10.LL* core enhancer perfectly co-segregates with the lobed-leaf phenotype, enabling efficient marker-assisted breeding of rapeseed.

**Supplementary Information:**

The online version contains supplementary material available at 10.1007/s44154-025-00280-3.

## Introduction

Rapeseed (*Brassica juncea* L., AABB, 2n = 36) exhibits distinct morphological characteristics and extensive genetic diversity. It is mainly cultivated in India, Pakistan, Africa, central Russia, Canada, Eastern Europe and the southwest and northwest regions of China. This species possesses multiple valuable traits, including drought resistance, poor soil tolerance, diseases and pests resistance, cold resistance, lodging resistance and pod shattering resistance (Kang et al. 2021).

Leaves are the primary organs of *B. juncea*, essential for photosynthesis, gas exchange, and water transport (Li et al. 2007). One of the main drivers of leaf morphological diversification is leaf margin variation (Tsukaya 2006). Leaf margin lobation is a leaf shape variation formed by plants to adapt to their living environments, which has important biological significance and is a key focus in the study of leaf morphology in *Brassicaceae* crops. Based on the depth of leaf margin lobation, leaves can be classified into several types, such as entire leaves, serrated leaves, and lobed leaves. Lobed leaves exhibit greater spatial extensibility, can regulate leaf surface temperature, and reduce water loss, thereby accumulating more photosynthetic products to adapt to harsh environments such as high temperature, cold, and drought (Tsukaya 2006; Vogel 2009). Therefore, identifying the genes controlling leaf margin lobation and elucidating their regulatory mechanisms will lay a solid foundation for the utilization of leaf shape traits in the genetic improvement of *B. juncea*.

In *Arabidopsis thaliana*, it was first demonstrated that the Homeobox-Leucine Zipper (HD-ZIP) transcription factor encoded by the *LATE MERISTEM IDENTITY 1* (*LMI1*) gene plays an important role in the formation of serrated leaves (Saddic et al. 2006). Through forward genetic screening of leaf shape in *Cardamine hirsuta*, the *REDUCED COMPLEXITY* (*RCO*) gene was successfully identified (Vlad et al. 2014). This gene is thought to have evolved via tandem duplication of the *LATE MERISTEM IDENTITY 1* (*LMI1*) gene and has no homolog in *A. thaliana*. The increased expression of *RCO* in *A. thaliana* increases leaf complexity, indicating that the loss of *RCO* during the process of speciation is the key to the simple leaf phenotype of this species (Vlad et al. 2014). However, unlike *LMI1*, *RCO* is specifically expressed at the base of leaflets. Through time-lapse imaging and growth analysis at the cellular level, it was found that *RCO* inhibits local growth at the base of leaflets (Vlad et al. 2014; Vuolo et al. 2016; Nikolov et al. 2019). There are three homologous copies of *LMI1-like* genes in *C. hirsuta*, namely *LMI1-like1*, *LMI1-like2* (*RCO*), and *LMI1-like3*. *RCO* promotes the formation of leaflets by inhibiting the growth of the leaf margin. In addition, the *LMI1-like* transcription factor plays an important role in the regulation of leaf shape in cotton. Silencing of *GhOKRA* results in wider cotton leaves, while its overexpression in *A. thaliana* enhances leaf lobation depth (Chang et al. 2016; Andres et al. 2017). *RCO* directly regulates genes involved in cytokinin (CK) homeostasis, and enhancing CK signaling within the *RCO* expression domain in *A. thaliana* increases leaf complexity (Hajheidari et al. 2019). In *Brassicaceae*, *RCO* does not affect auxin transport but cooperates with the CUC-auxin regulatory module to inhibit cell growth on both sides of the lobation, thereby modulating lobation depth (Vlad et al. 2014). More recently, *BnA10.RCO* was shown to positively regulates the formation of leaf margin lobation and overall leaf complexity in *Brassica napus* (Hu et al. 2020). However, the regulatory mechanism of the *RCO* gene underlying leaf shape formation in *B. juncea* remains unclear. Here, we employed two phenotypically distinct advanced inbred lines of *B. juncea* (the entire-leaf line 9B18 and the deeply lobed-leaf line 9B16), which exhibit stable and significant leaf morphological differences after multiple generations of selfing, making them ideal genetic resources for investigating leaf morphogenesis. We demonstrate that the *BjA10.LL* gene positively regulates leaf margin lobation in *B. juncea*. This finding not only provides novel insights into the molecular regulation of leaf shape but also offers a valuable candidate gene for molecular design breeding in rapeseed.

## Results

### Phenotypic and physiological characteristics of entire-leaf margins and lobed-leaf margins

In this study, two advanced inbred lines, 9B18 and 9B16, which exhibit significant differences in leaf margin morphology, were used as parental materials. An F_1_ population was generated by crossing these two lines. The results showed that 9B16 displayed deeply lobed leaves, while the hybrid F_1_ progeny exhibited serrated leaves, consistent with an intermediate phenotype of incomplete dominance (Fig. S1).

**Fig. 1:**
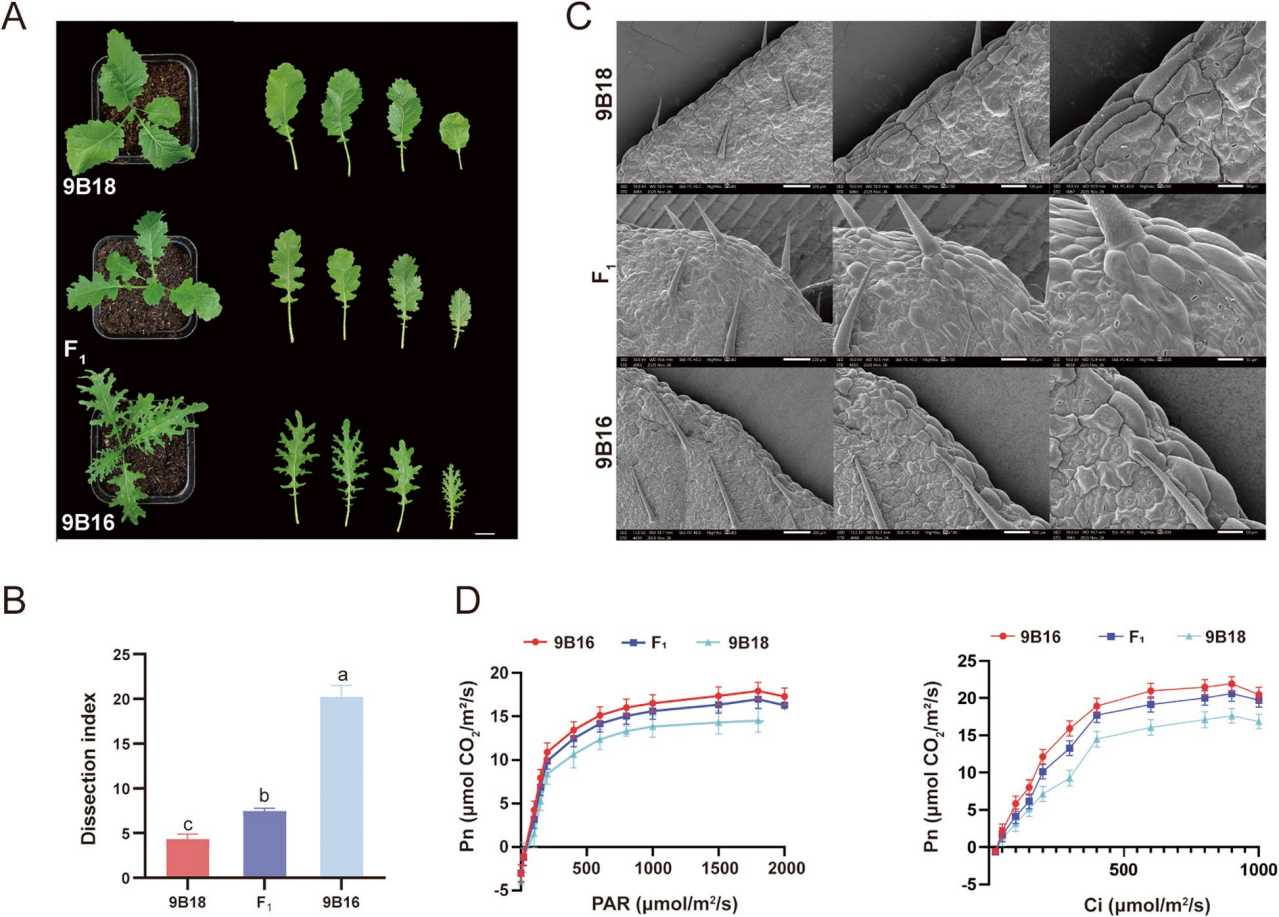
Phenotypic and physiological characteristics of the leaf margins in the two advanced-generation inbred lines 9B18, 9B16 and their F_1_ hybrid. A The lobed-leaf margin phenotypes of the leaves of 9B18, 9B16 and F_1_. 9B18 represents entire-leaf margins, 9B16 represents lobed-leaf margins. Scale bar = 2 cm. B Leaf dissection index of 9B18, 9B16 and F_1_ plants. Values are means ± SD (*n* = 10). Bars labeled with different lowercase letters indicate significant differences (*P* ≤ 0.01, one-way ANOVA followed by Tukey's HSD test). C Scanning electron microscopy observation of leaf margin cells in seedling leaves of 9B18, 9B16 and F_1_. Scale bars = 200 μm, 100 μm, and 50 μm. D Photosynthetically active radiation intensity and intercellular CO_2_ concentration between the leaves of 9B18, 9B16 and F_1_. Values are means ± SD (*n* = 10)

Further analysis of the phenotypic and physiological characteristics of leaf margins in two advanced inbred parental lines (9B18 and 9B16) and their F_1_ hybrid revealed that the lobed-leaf margin trait could be observed from the first true leaf stage throughout the entire growth period (Fig. 1A). Significant differences in leaf anatomical indices were observed between the entire-leaf parent and the lobed-leaf parent (Fig. 1B). The formation of lobed-leaf margins is attributed to the activity of meristematic tissues in specific regions of the leaf margin, which prolongs the undifferentiated development of the leaf and delays its maturation. To elucidate the relationship between leaf margin cell characteristics and leaf shape, we conducted detailed observations of the microscopic structures of leaf margins in entire-leaf 9B18, lobed-leaf 9B16 and F_1_ using scanning electron microscopy. The results showed that, compared to the typical elongated rod-shaped cells and larger protrusions at the leaf margins of 9B18, the leaf margins of 9B16 exhibited characteristics of meristematic cells, including smaller, rounder, and densely arranged cells (Fig. 1C). These findings suggest that cell division and growth processes resembling meristematic activity ultimately lead to the formation of the lobed-leaf margin trait in 9B16.

Additionally, we analyzed the impact of the lobed-leaf margin trait on photosynthetic efficiency. By measuring the photosynthetically active radiation intensity (PAR) and intercellular CO₂ concentration (Ci) in both parental lines (9B18 and 9B16) and their F_1_ hybrid, we found that 9B16 exhibited significantly higher PAR intensity and Ci compared to 9B18, with F_1_ progeny showing intermediate values (Fig. 1D). These results indicate that the lobed-leaf material demonstrated greater photosynthetic utilization efficiency than the entire-leaf material in this study.

### Inheritance of the lobed-leaf margin trait

To study the genetic pattern of the lobed-leaf margin trait, we constructed F_1_, F_2_ and BC_1_ segregating populations by crossing 9B18 (entire-leaf margin) and 9B16 (lobed-leaf margin). Based on two consecutive years of field observations in 2021 and 2022, the segregation of the target traits occurred in both the F_2_ and BC_1_ populations, which were manifested as entire-leaf margins and lobed-leaf margins respectively. Chi-square test showed that the segregation ratio of entire-leaf margin to lobed-leaf margin individuals in the F_2_ population fitted a 1:3 ratio, while the ratio in the BC_1_ population fitted 1:1. These results indicate that the lobed-leaf margin phenotypic trait of 9B16 is controlled by a single nuclear gene, without cytoplasmic genetic effects, and the lobed-leaf margin is an incompletely dominant trait (Table 1).

**Table 1:** Genetic analysis of the lobed-leaf margin trait

Years	Population	Plants tested	Entire-leaf	Lobed-leaf	Mendelian expectations	χ^2^	*P*
2021	9B18 (P_1_)	100	100	0	—	—	—
	9B16 (P_2_)	100	0	100	—	—	—
	F_1_	100	0	100	—	—	—
	F_2_	1813	465	1348	1:3	0.406	0.524
2022	9B18 (P_1_)	100	100	0	—	—	—
	9B16 (P_2_)	100	0	100	—	—	—
	F_1_	100	0	100	—	—	—
	BC_1_	787	403	384	1:1	0.459	0.498
	F_2_	469	127	342	1:3	1.081	0.299

### Fine mapping of the lobed-leaf margins gene

To rapidly localize the target gene region, this study employed the high-density *Brassica* 50 K Illumina Infinium™ SNP array, combined with bulked segregant analysis (BSA) and collinearity analysis, to screen for SNP markers and preliminarily map the leaf margin lobation gene in *B. juncea*. SNP array analysis identified four polymorphic SNP loci between 9B18 and 9B16 from genome-wide distributed SNP markers, all located on the A10 chromosome of *B. napus* (Table S2). Through collinearity comparison, the 17.07 ~ 17.38 Mb region on the A10 chromosome of *B. napus* was mapped to the 18.16 ~ 19.32 Mb region on the A10 chromosome and the 0.51 ~ 0.53 Mb region on the B02 chromosome of *B. juncea* (Table S3).

**Fig. 2:**
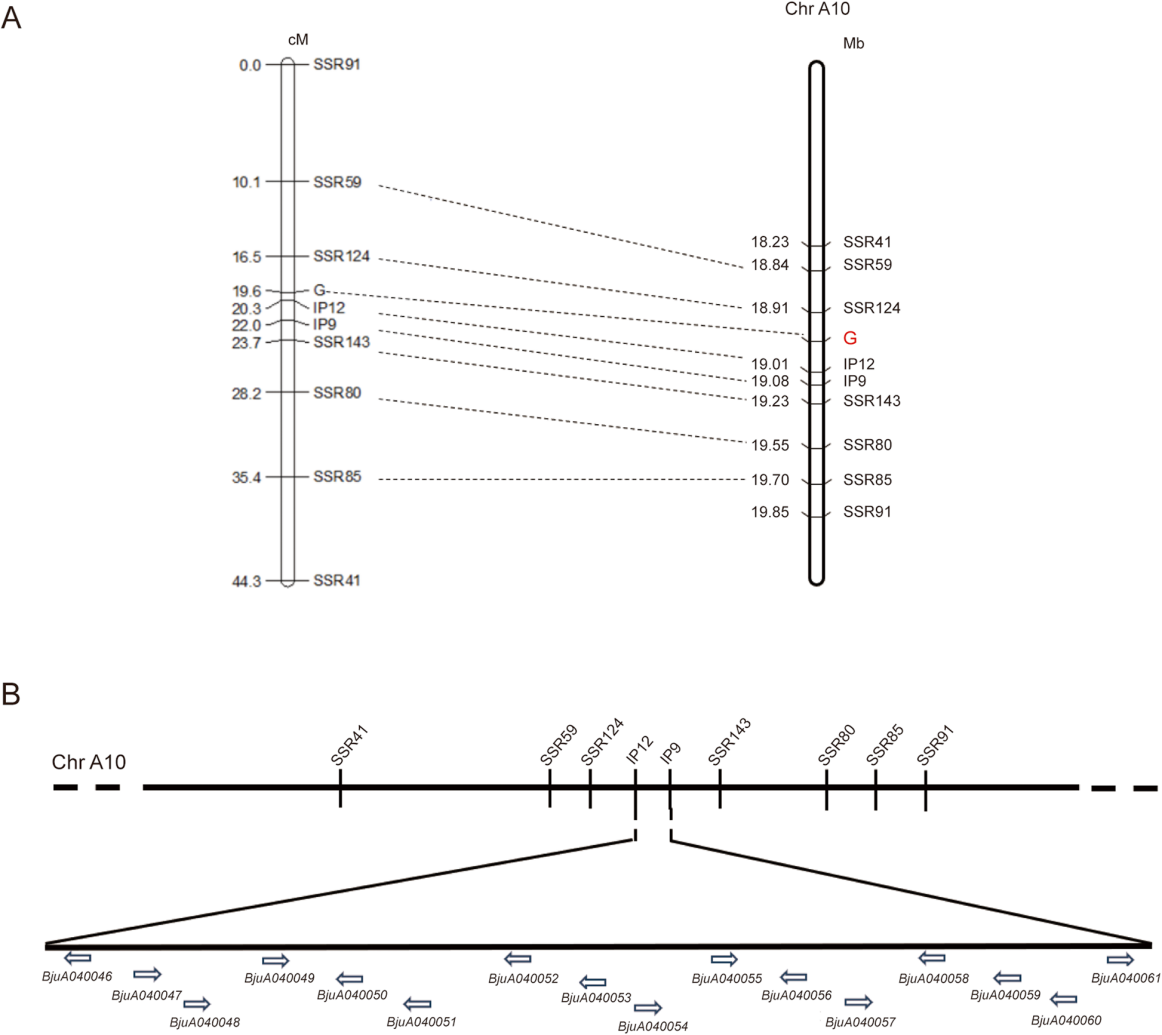
Fine mapping of the *BjA10.LL* genes. A Genetic map and physical map of *BjA10.LL* genes. B The distribution of 16 genes in the target interval

Within the target interval, 179 pairs of simple sequence repeat (SSR) primers and 17 pairs of intron polymorphism (IP) primers were designed. Among these, seven SSR primers (SSR41, SSR59, SSR80, SSR85, SSR91, SSR124, and SSR143) and two IP primers (IP9 and IP12) exhibited stable polymorphism in a small population (Fig. S2). These nine stable molecular markers were used to genotype 839 individuals from the F_2_ population, and the genetic distances between the markers and the target gene were calculated based on the number of recombinant individuals detected. Ultimately, the target gene was confined to a 3.8 cM region. The closest flanking markers, IP12 and SSR124, were located at genetic distances of 19,010,522 bp and 18,911,075 bp from the target gene, respectively, with a physical distance of 995 kb between them (Fig. 2A). The localization analysis revealed that the target interval encompasses a total of 16 genes (Fig. 2B). Functional annotation of these 16 genes was performed by integrating the *B. juncea* and *A. thaliana* databases (Table S4). Among these genes, only *BjuA040054* encodes an HD-ZIP I protein, which has been shown to play a significant role in the formation of leaf margin lobation (Hu et al. 2020).

### Sequence alignment and transcriptome analysis identified *BjA10.LL* as a candidate gene

The amino acid sequences of the candidate gene *BjuA040054* and the *RCO* gene in *B. napus* were aligned using the DNAMEAN software (Fig S3). The results showed that the protein encoded by *BjuA040054* shares high sequence identity with RCO protein (BnaA10g26320D) from *B. napus*. This indicates that the proteins encoded by the *BjuA040054* gene in *B. juncea* and the *RCO* gene in *B. napus* may have similar biological functions.

**Fig. 3:**
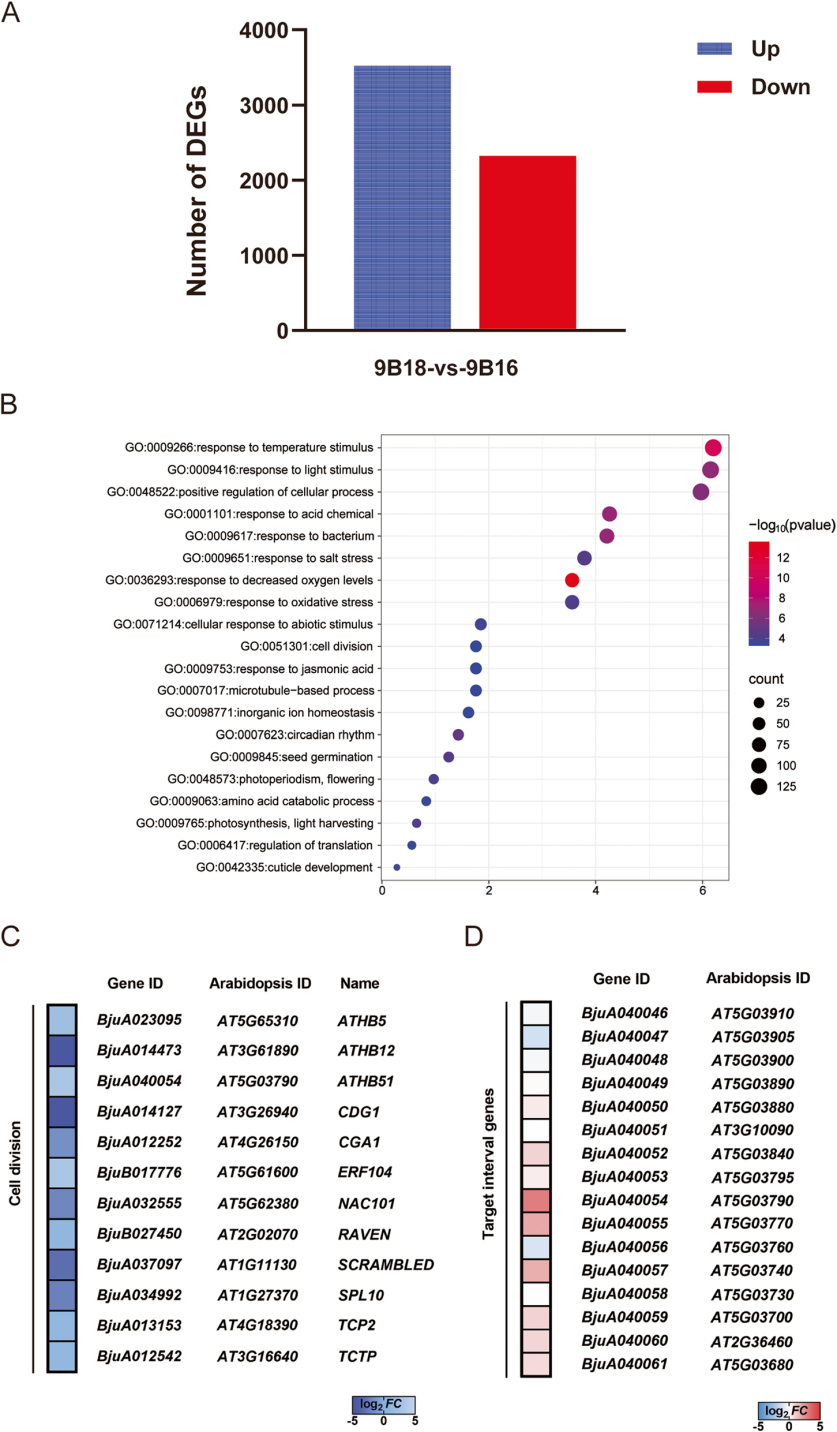
Transcriptome analysis of *BjA10.LL* regulation. A Statistics of differentially expressed genes. B Differential expression genes GO enrichment. C Gene expression analysis related to cell differentiation. D Expression analysis of candidate genes within the target interval. The numerical values in the legend represent the log_2_ fold change value of the expression level of this gene in 9B16 compared to that in 9B18

To further validate the candidate gene and explore the molecular mechanism of the lobed-leaf margin formation in *B. juncea*, we used entire-leaf plants (9B18) as the control group and lobed-leaf plants (9B16) as the treatment group, and performed transcriptome sequencing on the leaves at the five-leaf stage. For differential expression analysis, |Log_2_ (Fold Change)|≥ 2 and FDR < 0.05 were set as the screening criteria. The analysis results showed that a total of 5846 differentially expressed genes (DEGs) were identified, among which 3523 genes were up-regulated and 2323 genes were down-regulated (Fig. 3A). To explore the biological functions of the DEGs related to the formation of lobed-leaf margins in this study, we used the BLAST software for sequence alignment to obtain the homologous genes of each DEGs in *A. thaliana*, and then conducted GO enrichment analysis. The biological processes that were significantly and commonly enriched include cell division, photosynthesis, photoperiodism, positive regulation of cellular process, etc.(Fig. 3B). Meanwhile, based on the GO annotation information, we further analyzed the DEGs related to cell division. Among them, genes such as *ATHB5*, *ATHB51*, *TCP2*, and *TCTP* were upregulated, while genes such as *CDG1*, *SPL12*, and *NAC101* were downregulated (Fig. 3C). To verify the authenticity of the RNA sequencing (RNA-seq) data, we randomly selected nine DEGs for RT-qPCR verification. The results showed that the expression trends of these genes in 9B18 and 9B16 were consistent with the results of transcriptome sequencing (Fig. S4), confirming the reliability of the transcriptome sequencing data.

By integrating transcriptome and gene mapping results, it was found that among the 16 genes in the mapped candidate interval, only *BjuA040054* was differentially expressed. In conclusion, we speculate that *BjuA040054* (designated as *BjA10.LL*) is the candidate gene controlling the lobed-leaf margin trait in *B. juncea*.

### *BjA10.LL* expression pattern and subcellular localization

**Fig. 4:**
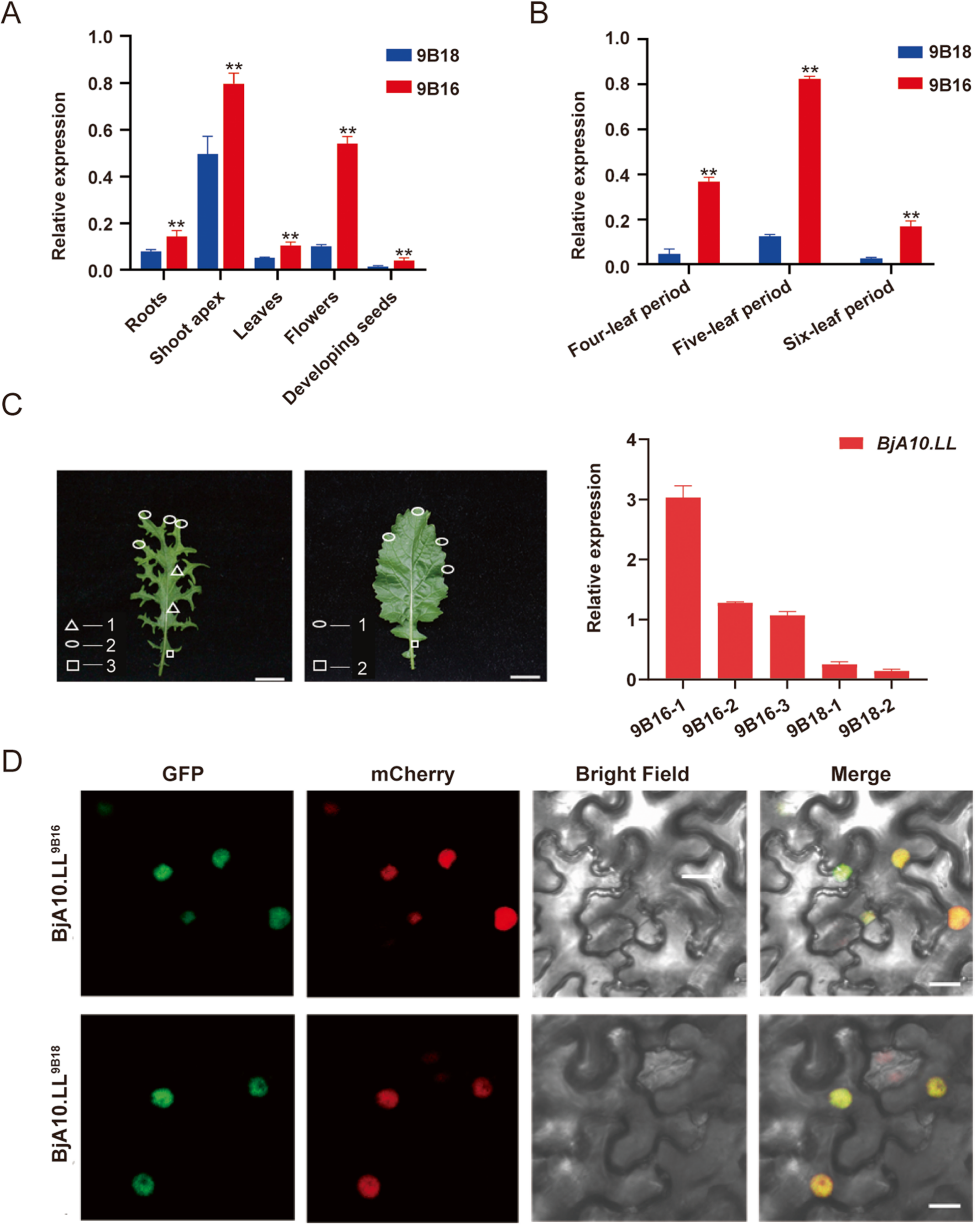
Analysis of the *BjA10.LL* expression pattern. A RT-qPCR analysis of the expression of *BjA10.LL* in different tissues of *B. juncea*. Scale bars: 1 cm. B RT-qPCR analysis of *BjA10.LL* in the leaves of 9B18 and 9B16 seedlings at the four-leaf period, five-leaf period and six-leaf period. C RT-qPCR analysis of 9B18 and 9B16 in different leaf segments at the five-leaf period. D Subcellular localization of BjA10.LL^9B18^ and BjA10.LL^9B16^ proteins fused with green fluorescent protein (GFP) (*35S:BjA10.LL*.^*XX*^*–GFP*) in tobacco leaves. mCherry is a nuclear localized protein fused with a red fluorescent protein; merge, merge of mCherry, GFP, and bright field images. Scale bars = 50 μm. Results were normalized against the expression of *BjuUBQ9* as an internal control. Values are means ± SD (*n* = 3). Double asterisks indicate that there are highly significant differences in the expression levels between 9B18 and 9B16 (two-tailed paired Student's *t*-test, *P* ≤ 0.01). The ‘*XX*’ of *BjA10.LL* means 9B18 and 9B16

To study the function of *BjA10.LL*, we analyzed its spatiotemporal expression patterns in 9B18 and 9B16 using RT-qPCR. The results showed that *BjA10.LL* is widely expressed in various tissues, with particularly high expression in the shoot apex (Fig. 4A). Additionally, its expression level was highest at the five-leaf stage (Fig. 4B). Furthermore, we investigated the detailed expression patterns of *BjA10.LL* in different leaf parts at the five-leaf stage in 9B18 and 9B16. The results revealed that in 9B18, *BjA10.LL* expression was relatively low in all leaf parts, whereas in 9B16, it was expressed in nearly all leaf parts at relatively high levels (Fig. 4C).

To determine whether *BjA10.LL* functions as a transcription factor, we observed the GFP fluorescence signal of the fusion protein BjA10.LL^XX^–GFP expressed in tobacco leaves. The results showed that both *35S:BjA10.LL*^*9B18*^*–GFP* and *35S:BjA10.LL*^*9B16*^*–GFP* were specifically localized in the nucleus (Fig. 4D). These results suggest that *BjA10.LL* may act as a transcription factor to regulate leaf development in *B. juncea*.

### Functional analysis of *BjA10.LL* in regulating the formation of leaf margin lobes in *A. thaliana*

To investigate the role of the *BjA10.LL* gene in the formation of lobed-leaf margins, we amplified the DNA fragments of *BjA10.LL* from the 9B18 and 9B16 lines, respectively. Comparative analysis revealed three nonsynonymous SNP differences between the two parental lines (Fig. S5A). Further amplification of the cDNA fragments from 9B18 and 9B16 showed that, at the 35th bp position of the first exon, the nucleotide sequence changed from ACG to ATG, resulting in the substitution of threonine (Thr) with methionine (Met) at the 12th amino acid position (Fig. S5B).

**Fig. 5:**
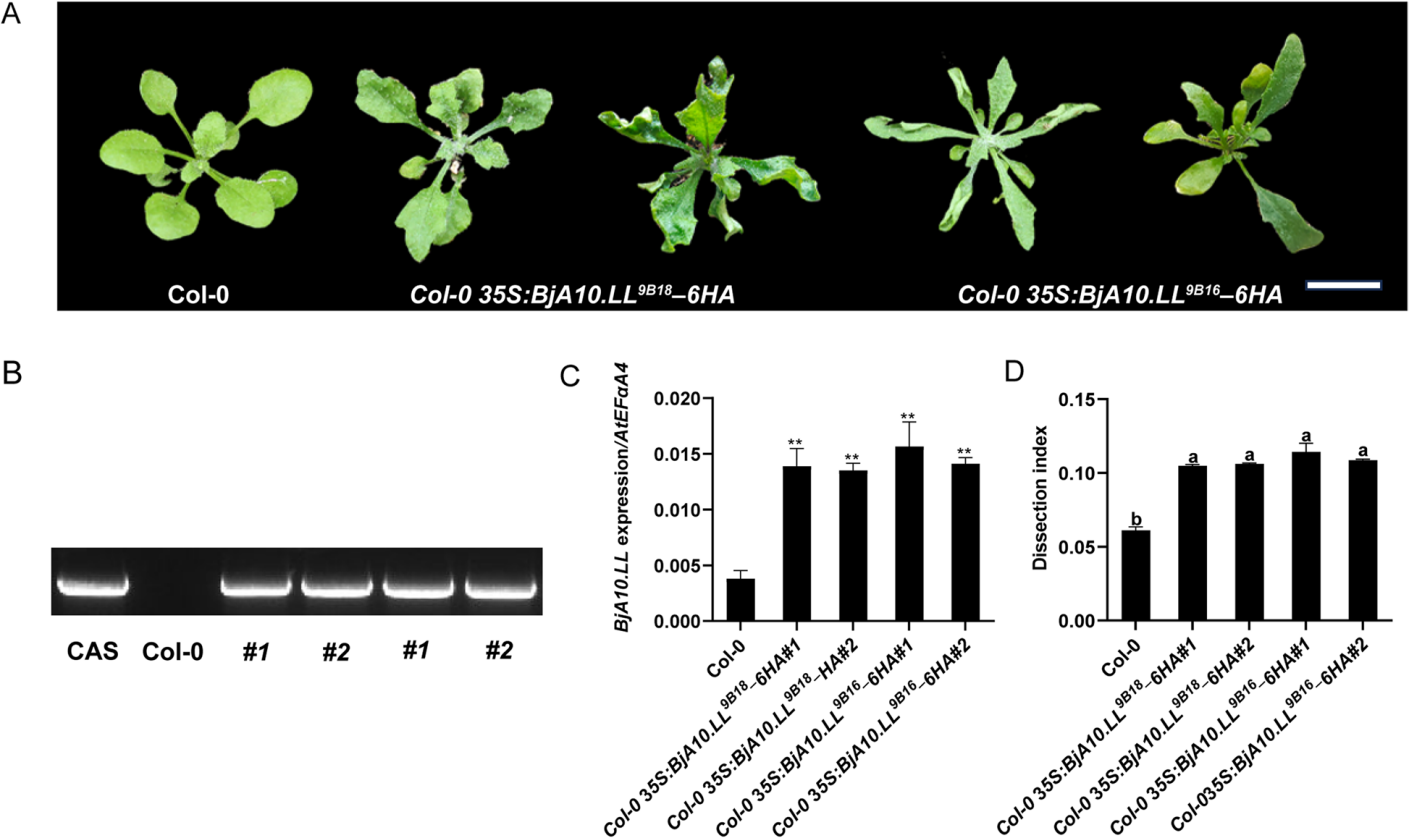
Functional analysis of *BjA10.LL* in the formation of leaf lobes in *A. thaliana.* A Leaf phenotypes of the *35S:BjA10.LL*^*XX*^*–6HA* overexpression transgenic and the wild type *A. thaliana* plants (Col-0). Scale bar: 1 cm. B PCR-based DNA genotyping of *35S:BjA10.LL*^*XX*^ transgenic plants. Cas, cassette. C The expression levels of *BjA10.LL* in the fifth true leaves of the *35S:BjA10.LL*^*XX*^*–6HA* overexpression transgenic and the wild type *A. thaliana* plants (Col-0). Values are means ± SD (*n* = 3). Double asterisks indicate that there are highly significant differences in the expression levels of the fifth true leaves between the *35S:BjA10.LL*^*XX*^*–6HA* overexpression transgenic and the wild type *A. thaliana* plants (two-tailed paired Student's *t*-test, *P* ≤ 0.01). D The leaf dissection index of the fifth true leaves of the *35S:BjA10.LL*.^*XX*^*–6HA* overexpression transgenic and the wild type *A. thaliana* plants (Col-0). The ‘*XX*’ of *BjA10.LL* means 9B18 and 9B16

In order to further explore whether the changes in amino acids affect the formation of leaf margin lobation, we transformed the overexpression constructs of *35S:BjA10.LL*^*9B18*^*–6HA* and *35S:BjA10.LL*^*9B16*^–*6HA* into *A. thaliana* Col-0 plants, respectively. Through hygromycin selection, 10 independent T_1_ transgenic lines were obtained. The expression level of *BjA10.LL* in the representative overexpression transgenic lines of the T_3_ generation was measured by RT-qPCR. The results showed that, compared with the wild type, the expression of *BjA10.LL* in the transgenic lines was significantly up-regulated. All the transgenic lines produced deeply incised leaves that had never been observed in the wild type (Fig. 5A-D). These results indicate that *BjA10.LL* positively regulates the formation of leaf margin lobation in *A. thaliana* leaves.

### Promoter activity assays of *BjA10.LL*

To gain deeper insights into the function of *BjA10.LL* and investigate whether sequence variations in its promoter region are associated with the formation of lobed-leaf margins, we cloned ~ 3 kb promoter fragments of *BjA10.LL* from both parental lines. Sequence alignment revealed significant differences in this region between 9B18 and 9B16 (Fig. S6).

**Fig. 6:**
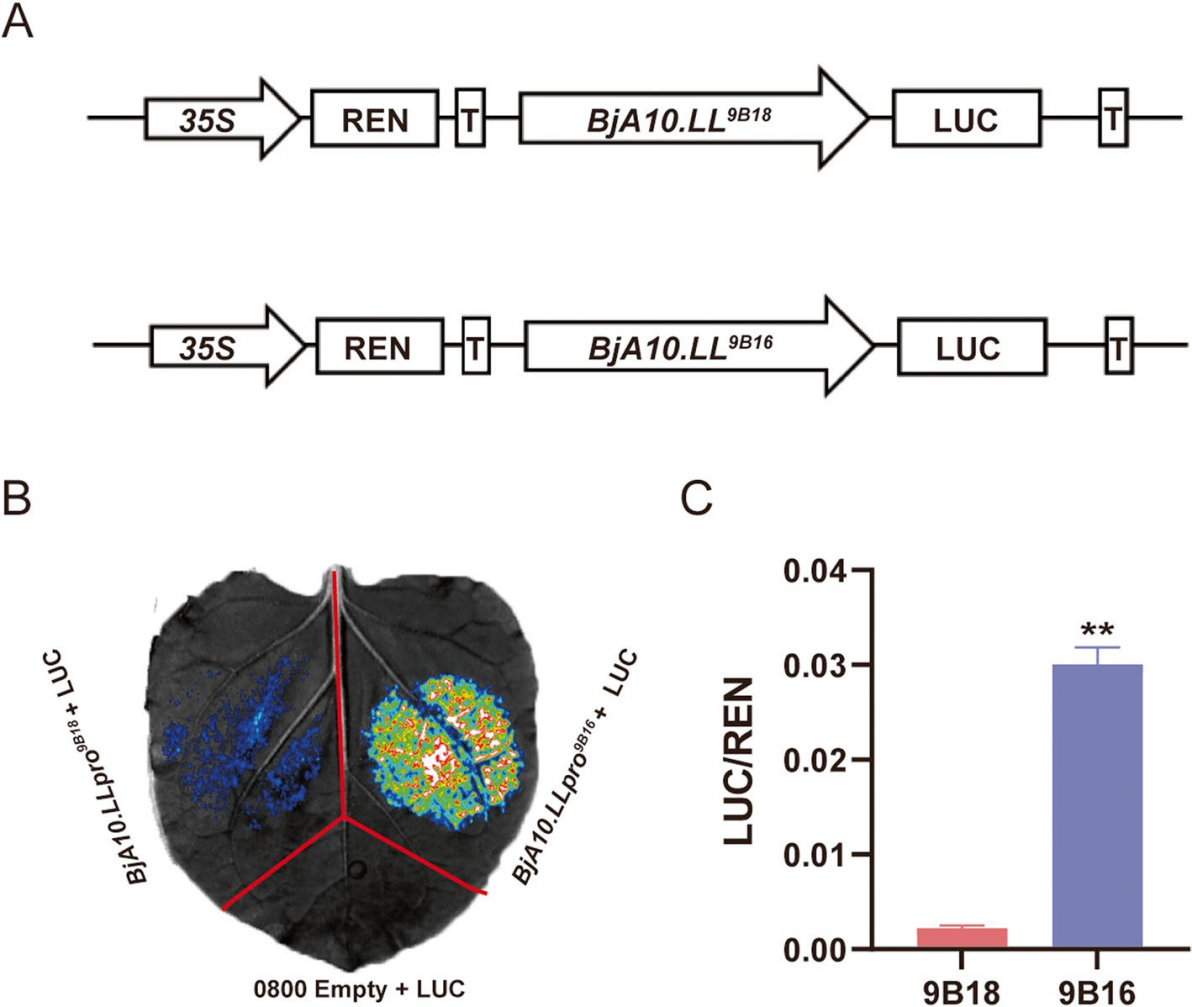
Transient dual luciferase (dual-LUC) assays of *BjA10.LL*^*9B18*^ and *BjA10.LL*^*9B16*^ in tobacco leaves. A Sequence alignments of the ~ 3.0 kb region upstream of the start codon of *BjA10.LL* in 9B18 and 9B16. B Activity assays of the promoters of *BjA10.LL*^*9B18*^ and *BjA10.LL*.^*9B16*^ based on the dual luciferase system. C Quantification of the dual-luciferase assay. The relative LUC/REN activity ratio confirms that the lobed-leaf haplotype (9B16) possesses significantly stronger enhancer activity than the entire-leaf haplotype (9B18). Data are presented as mean ± SD (n = 5) (two-tailed paired Student's *t*-test, *P* ≤ 0.01)

To explore whether the sequence variations in the *BjA10.LL* promoter are responsible for the formation of leaf margin lobes, the promoter activities of *BjA10.LL* in 9B18 and 9B16 were tested via transient transcriptional activity assays. As shown, the promoter activity of *BjA10.LL*^*9B16*^ was significantly higher than that in *BjA10.LL*^*9B18*^ (Fig. 6).

**Fig. 7:**
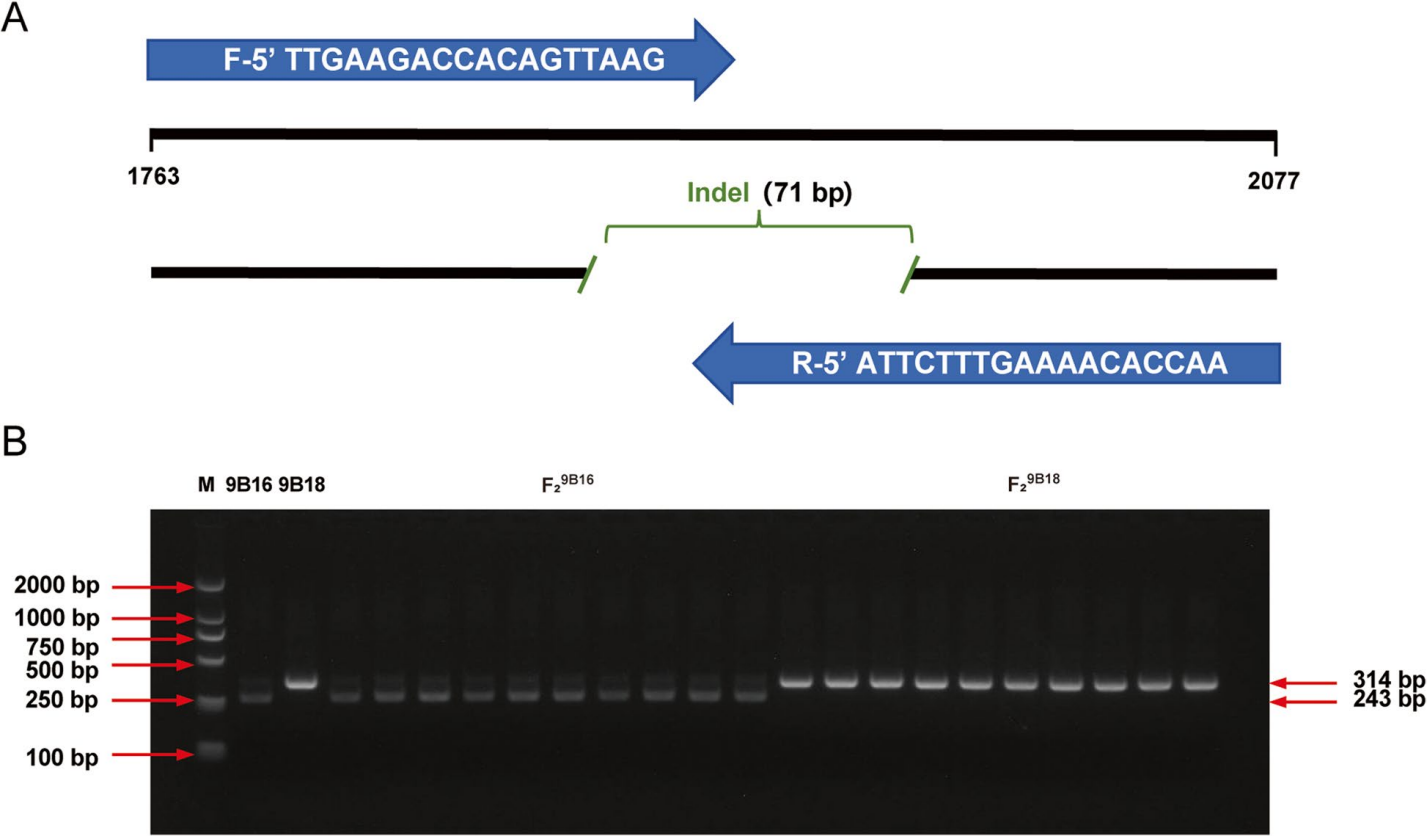
Genotyping with the co-dominant molecular marker developed in the ~ 3.0 kb region upstream of the *BjA10.LL* start codon. A Schematic diagram of molecular marker-specific primers and corresponding sequence structure. B Genotyping results revealed by PCR amplification in the parental lines (9B16 and 9B18) and a subset of F_2_ plants displaying distinct leaf phenotypes. The PCR products were separated on a 2% agarose gel. M: Marker

To further validate that the sequence variation within this enhancer region is the causal polymorphism underlying natural variation in leaf morphology, we developed a co-dominant molecular marker. Primers were designed to flank two major indels within the ~ 500 bp enhancer of *BjA10.LL*, which effectively distinguished the parental haplotypes. Genotyping of the segregating F_2_ population revealed a perfect co-segregation between the marker haplotype and the leaf margin phenotype (Fig. 7). All plants carrying the lobed-leaf haplotype exhibited lobed leaves, whereas those for the entire-leaf haplotype displayed entire leaves.

This tight genetic linkage, together with the differential transcriptional activity demonstrated by the dual-luciferase assay, provides compelling evidence that the structural variation in the *BjA10.LL* enhancer region is responsible for the differential gene expression and, consequently, the divergence in leaf shape between the two parental lines.

## Discussion

HD-ZIP I proteins have been demonstrated to play a crucial role in leaf development. For instance, transgenic plants overexpressing *AtHB23*, *AtHB3*, *AtHB13*, and *AtHB20* exhibit significant changes during leaf growth and development (Wang et al. 2003; Henriksson et al. 2005; Kim et al. 2007). In *B. napus*, previous studies have identified that the HD-ZIP I transcription factors *BnA10.RCO* and *BnA10.LMI1* are involved in regulating the formation of leaf margin lobation. In this study, we performed amino acid sequence alignment of the candidate gene *BjA10.LL* with these genes, revealing that only *BnA10.RCO* shares high homology with *BjA10.LL*. Functional annotation of the *A. thaliana* homologous gene further confirmed that *BjA10.LL* encodes an HD-ZIP I protein, consistent with previous findings.

Previous studies have shown that the *RCO* gene (but not *LMI1*) plays a pivotal role in leaf morphological development in *Arabidopsis lyrata*, *C. hirsuta*, and *B. napus* (Sicard et al. 2014; Vlad et al. 2014; Heng et al. 2020; Hu et al. 2020). In this study, we identified significant cis-regulatory differences between the parental alleles of *BjA10.LL*. Consistent with this finding, the promoter activity of *BjA10.LL*^*9B16*^ was significantly enhanced in the incised-leaf parent compared to the entire-leaf parent. Additionally, we observed variations in the enhancer region of the lobed-leaf parent, including six SNPs, one insertion, and two deletions. To genetically link these sequence variations to the phenotype, we developed a co-dominant molecular marker targeting two major indels within a core ~ 500 bp enhancer region. This marker exhibited perfect co-segregation with the leaf margin phenotype in our segregating F_2_ population, providing strong genetic evidence that these enhancer variations are indeed the causal polymorphisms responsible for differential gene expression and the consequent leaf shape determination. mVISTA alignment analysis of the *RCO* gene (*BjA10.LL*) in *B. juncea* with those in *B. napus* and *C. hirsuta* revealed high sequence conservation, including a conserved B region (Fig. S7). These results suggest that the function of the *RCO* gene in *B. juncea* may be similar to that in *C. hirsuta*. To validate this hypothesis, future studies should truncate the *RCO* gene and construct promoter deletion fragments of varying lengths combined with a reporter gene system to investigate the regulatory function of the B region on *RCO* gene expression. This research will help elucidate the expression regulation mechanism of the *RCO* gene in *B. juncea* and provide important insights into its molecular function in leaf morphogenesis.

Cytokinins, as one of the five major classes of plant hormones, play a crucial regulatory role in the growth and development of higher plants. Research has demonstrated that cytokinins are not only involved in regulating cell division and differentiation but also play key roles in various physiological processes, including organogenesis, apical dominance maintenance, and leaf senescence delay (Miyawaki et al. 2004). Particularly in the complex process of leaf margin development, cytokinins exert significant influence on leaf morphogenesis by maintaining the activity of meristematic tissues.

From a molecular perspective, the biosynthesis and metabolism of cytokinins are precisely regulated by multiple key genes. Among these, *IPT7* is a critical gene in the cytokinin biosynthesis pathway. Studies in tomato (*Solanum lycopersicum*) have shown that overexpression of the *A. thaliana AtIPT7* gene significantly increases cytokinin levels, thereby promoting leaf morphological complexity and leading to more pronounced serrated leaf margins (Dello Ioio et al. 2012). This finding has been further validated by reverse genetics experiments: when the cytokinin degradation gene *CKX3* is heterologously expressed, cytokinin levels are significantly reduced, resulting in the disappearance of leaf lobation (Shani et al. 2010; Bar et al. 2017). In *C. hirsuta*, enhanced cytokinin activity in the *RCO* gene region has been shown to significantly promote the formation of leaf complexity (Hajheidari et al. 2019). These results collectively confirm the positive regulatory role of cytokinins in the formation of leaf margin lobation. Notably, the regulation of leaf margin morphology by cytokinins may involve a complex signaling network. Early research by Rupp et al. (1999) demonstrated that cytokinins significantly promote the expression of the *KNOX* family gene *STM*, which has been shown to enhance the development of complex leaf margins. In this study, we conducted transcriptome sequencing analysis on the entire-leaf margin parent 9B18 and the lobed-leaf parent 9B16, followed by GO functional enrichment analysis of differentially expressed genes. The results revealed significant enrichment of genes related to cytokinin biosynthesis, metabolism, and signal transduction. This finding further supports the potential key role of cytokinins in regulating leaf margin lobing morphogenesis.

Based on these results, future research could further explore the molecular mechanisms of cytokinins in leaf lobe formation, particularly their interactions with the regulatory network of the *RCO* gene. This will provide new theoretical insights and research directions for understanding the genetic basis of plant leaf morphological diversity.

## Conclusion

This study revealed through genetic analysis that the lobed-leaf margin phenotype in *B juncea* is controlled by a single gene and exhibits incomplete dominance. Using map-based cloning techniques, we identified BjA10.LL as the key gene regulating the formation of lobed-leaf margins in *B. juncea*. Promoter activity assays demonstrated its positive regulatory role in this trait. Corresponding to the gene’s core enhancer variations, a co-dominant molecular marker targeting these indels was developed, which perfectly co-segregates with the phenotype and thus facilitates marker-assisted breeding of *B. juncea*.

## Materials and methods

### Plant materials

For the gene mapping of the lobed-leaf margin gene *BjA10.LL* in *B. juncea*, a F_1_, F_2_, BC_2_ segregation population was constructed using parental lines of *B. juncea* with entire-leaf margins (9B18) and lobed-leaf margins (9B16). These materials were planted at the experimental farm of Northwest A&F University (Yangling, Shaanxi, China) in the middle and late September each year from 2021 to 2024. Field management followed standard agricultural practices. At the five-leaf stage, the leaf types of both parental materials and the mapping population were recorded. Leaves were collected from individual plants, flash-frozen in liquid nitrogen, and transported to the laboratory for DNA extraction. The extracted DNA was used for subsequent gene mapping and cloning analyses. The parental lines (9B18 and 9B16) and their F_1_ hybrids were grown in a growth chamber at 22 °C under a long day duration (16 h light/8 h dark). Phenotypic observations were conducted at the five-leaf stage, and leaves from 20-day-old seedlings were sampled for scanning electron microscopy (SEM) analysis. *A. thaliana* (Col-0) was used as the wild type control in this study. All *A. thaliana* plants were grown in a growth chamber at 22 °C under long-day conditions. Tobacco plants were incubated in a growth chamber at 22 °C with a 16 h light/8 h dark photoperiod for 72 h.

### Trait measurement

In order to quantify the degree and complexity of leaf lobing, the leaf was calculated according to (perimeter-squared)/(4π × area) (Hu et al. 2018).

To clarify the relationship between leaf margin cell characteristics and leaf shape, scanning electron microscopy was used to observe the leaf margin cells. Leaves smaller than 0.2 mm were collected from 20-day-old seedlings and fixed in FAA fixative solution for over 12 h. The samples were then dehydrated 1 to 2 times using a series of graded ethanol solutions, with each solution volume being at least three times that of the samples. After dehydration, the materials were dried using the critical point drying method. Subsequently, the dried samples were mounted onto copper stages with conductive adhesive, sputter-coated with gold, and observed under a scanning electron microscope (TM4000 Plus).

To evaluate the impact of the lobed-leaf margin trait on photosynthetic characteristics, PAR and Ci of 9B16 and 9B18 were measured in the field. The fifth leaves of 9B18 and 9B16 under consistent growth conditions were analyzed using a portable photosynthesis system (LI-6400XT; Li-COR Inc., Lincoln, NE, USA). Measurements were conducted between 9:00 and 11:00 in the morning (Sun et al. 2016). Three biological replicates were used, with each replicate consisting of 10 plants.

### DNA extraction and SNP genotyping

For single-nucleotide polymorphism (SNP) genotyping (*Brassica* 50 K Illumina Infinium™ SNP), total genomic DNA was extracted from fresh leaves of the two parents (10 individual plants each for 9B18 and 9B16) and the F₂ population plants, including 30 plants with entire-leaf margins and 30 plants with lobed-leaf margins, using a plant genomic DNA extraction kit (Tiangen Biotech, Beijing, China) following the manufacturer’s protocol. Each bulk DNA sample was constructed with equivalent amounts of DNA from ten individuals of each parent and 30 individuals from the F₂ population. Finally, four bulk DNA samples, including the two parents, F₂ entire-leaf margin plants, and F_2_ lobed-leaf margin plants, were used for SNP genotyping with a *Brassica* 50 K Illumina Infinium™ SNP array, according to the method described by Xiao et al. (2021). Meanwhile, the gene sequences were subjected to BLAST alignment, and the resulting data were analyzed for synteny using TBtools software.

### Molecular marker development, linkage analysis and locus mapping

Based on the genome assemblies of the *Brassica* database (BRAD: http://brassicadb.cn/#/) (Wang et al. 2011; Chen et al. 2022), molecular markers including SSR and IP in the *BjA10.LL* locus region, were developed. The SSR primers were designed using the web-based SSR finder tool (http://www.geboc.org/index/) Meanwhile, the IP primers were designed using Primer 5.0 software (http://www.premierbiosoft.com/primerdesign/). For functional characterization of *BjA10.LL*, specific primers were designed for promoter and CDS cloning. The sequence information of candidate genes is provided by the BRAD (http://brassicadb.org/brad/) and TAIR (http://www.arabidopsis.org) databases. Primers were designed based on standard criteria using the Primer-BLAST suite on the NCBI platform (https://www.ncbi.nlm.nih.gov/tools/primer-blast/). The resulting primer sequences were commercially synthesized by Sangon Biotech (Shanghai, China). The primers used are detailed in Supplemental Table S1.

### Candidate gene analysis

Extract the gene annotation information within the 995 kb interval of *BjA10.LL* from the *Brassica* database and the TAIR database. We further screened the candidate genes by analyzing the transcriptome data between the two parents. Using the *Brassica* database (http://brassicadb.org/brad/) as the reference genome, the following bioinformatics analysis pipeline was employed, including sequencing data quality assessment, reference sequence alignment analysis, gene expression analysis, screening of DEGs, and Gene Ontology (GO) enrichment analysis of the DEGs. During the screening of differentially expressed genes, the criteria were set as |Log_2_ (Fold Change)|≥ 2 and *FDR* < 0.05. The screened DEGs were subsequently submitted to the Bioinformatics Online Tool (https://www.bioinformatics.com.cn/) for GO enrichment analysis, and significantly enriched GO terms were identified. Finally, we predicted the candidate genes by combining sequence alignment analysis, transcriptome analysis and the functional annotations of the *Arabidopsis* homologs of the candidate genes.

### Sequence alignment analysis of *BjA10.LL*

The amino acid sequences of candidate genes and their homologous genes were retrieved from the *B. juncea* database (BRAD: http://brassicadb.cn/#/) and the *B. napus* database (BnIR: http://yanglab.hzau.edu.cn/), respectively. Sequence alignment was then performed using DNAMAN software.

### RNA extraction and quantitative real‑time PCR (RT‑qPCR) analysis

Total RNA samples were isolated from various vegetative tissues of plants, leaves at different stages, and different segments of the fifth leaf of parents during the rosette stage using the MiniBEST Plant RNA Extraction Kit (TaKaRa Bio, Dalian, China). Subsequently, the RNA was reverse-transcribed using PrimeScript RT Mix (TaKaRa Bio, Dalian, China). Quantitative real-time PCR was performed with three biological replicates using SYBR Green Master Mix (Cofitt, Hong Kong, China). *BjuUBQ9* and *AtEF1aA4*, ubiquitously expressed genes, were used as internal controls. All measurements were conducted with three independent biological replicates and two technical replicates. The primers used for gene expression analysis are listed in Table S1.

### Subcellular localization analysis of BjA10.LL

The *35S:BjA10.LL*^*9B18*^*–GFP* and *35S:BjA10.LL*^*9B16*^*–GFP* constructs were separately transformed into *Agrobacterium tumefaciens* strain GV3101 and transiently expressed in the young leaves of the transgenic *tobacco* with nuclear localization signal as previously described (Yang et al. 2010).

### Plant transformation

The constructs of *35S:BjA10.LL*^*9B18*^*–6HA* and *35S:BjA10.LL*^*9B16*^–*6HA* were transformed into the *A. thaliana* wild type (Col-0) separately using the floral dip method mediated by *A. tumefaciens* strain GV3101 to generate transgenic *A. thaliana* plants (Clough and Bent 2008).

### Promoter activity analysis of *BjA10.LL*

To analyze the promoter activity of *BjA10.LL*, specific amplification primers were designed. Approximately 3 kb promoter sequences of *BjA10.LL* were cloned from the genomic DNA of 9B18 and 9B16. The amplified products were subsequently inserted into the pGreenII 0800–LUC vector, generating the recombinant constructs. These constructs were then transferred into *A. tumefaciens* strain GV3101 with the pSoup-P19 (Weidi Biotechnology, Shanghai, China). The *Agrobacterium* culture was mixed with the recombinant vectors at a volume ratio of 1:1 and co-infiltrated into the young leaves of 4-week-old tobacco plants. After 72 h of cultivation, the infiltrated leaves were collected, and LUC signals were detected using a low-light cooled charge-coupled device imaging apparatus (Tanon 4600, Shanghai, China). Firefly LUC and Renilla luciferase (REN) activities were examined using the dual-luciferase reporter assay kit (YEASEN, Shanghai, China) according to the instructions. Five independent biological samples were assayed. The experiment was performed with five independent biological replicates, each consisting of three technical replicates.

## Supplementary Information

Additional file 1: Fig. S1 The characteristics of the leaf lobes of entire-leaf blades (9B18), lobed-leaf blades (9B16) and their F_1_ hybrid leaves. Fig. S2 Verification results of molecular markers in the parental plants and individual plants with entire and lobed leaves in the F_2_ generation. Fig. S3 Alignment of amino acid sequences of candidate genes. Fig. S4 RT-qPCR validation of some differentially expressed genes in the transcriptome. Fig. S5 Sequence analysis of *BjA10.LL*. Fig. S6 Sequence alignments of the ~ 3.0 kb upstream of the start codon of *BjA10.LL* in 9B18 and 9B16. Fig. S7 mVISTA graph of the alignment of the upstream sequence of the *BjA10.LL* gene in *B. juncea* with the *RCO* genes in *B. napus* and of *C. hirsuta*.

Additional file 2: Table S1. Sequences of primers. Table S2. Polymorphism SNP_S_ between the entire-leaf samples and the lobed-leaf samples obtained by *Brassica* 50K Illumina Infnium™. Table S3. Collinearity analysis of chromosomal locations. Table S4. The information of 16 genes in the target interval.

## Data Availability

All data included in this study are available from the corresponding author upon reasonable request.

## Abbreviations

CK: Cytokinin
SSR: Simple sequence repeat
IP: Intron polymorphism
PAR: Photosynthetically active radiation intensity
Ci: Intercellular CO₂ concentration (Ci)
DEGs: Differentially expressed genes
GO: Gene Ontology
RT-qPCR: Quantitative real‑time PCR
RNA-seq: RNA sequencing
