# Supplementary material for: Cloning and functional analysis of the lobed-leaf gene BjA10.LL in Brassica juncea L
Source: Stress Biol. 2026 Jan 20;6(1):8. doi: 10.1007/s44154-025-00280-3 (PMC12816491; doi:10.1007/s44154-025-00280-3)
Supplement: Supplementary file 1 — Additional file 1: Fig. S1 The characteristics of the leaf lobes of entire-leaf blades (9B18), lobed-leaf blades (9B16) and their F1 hybrid leaves. Fig. S2 Verification results of molecular markers in the parental plants and individual plants with entire and lobed leaves in the F2 generation. Fig. S3 Alignment of amino acid sequences of candidate genes. Fig. S4 RT-qPCR validation of some differentially expressed genes in the transcriptome. Fig. S5 Sequence analysis of BjA10.LL. Fig. S6 Sequence alignments of the ~ 3.0 kb upstream of the start codon of BjA10.LL in 9B18 and 9B16. Fig. S7 mVISTA graph of the alignment of the upstream sequence of the BjA10.LL gene in B. juncea with the RCO genes in B. napus and of C. hirsuta. [file 44154_2025_280_MOESM1_ESM.docx]

Supplementary Information


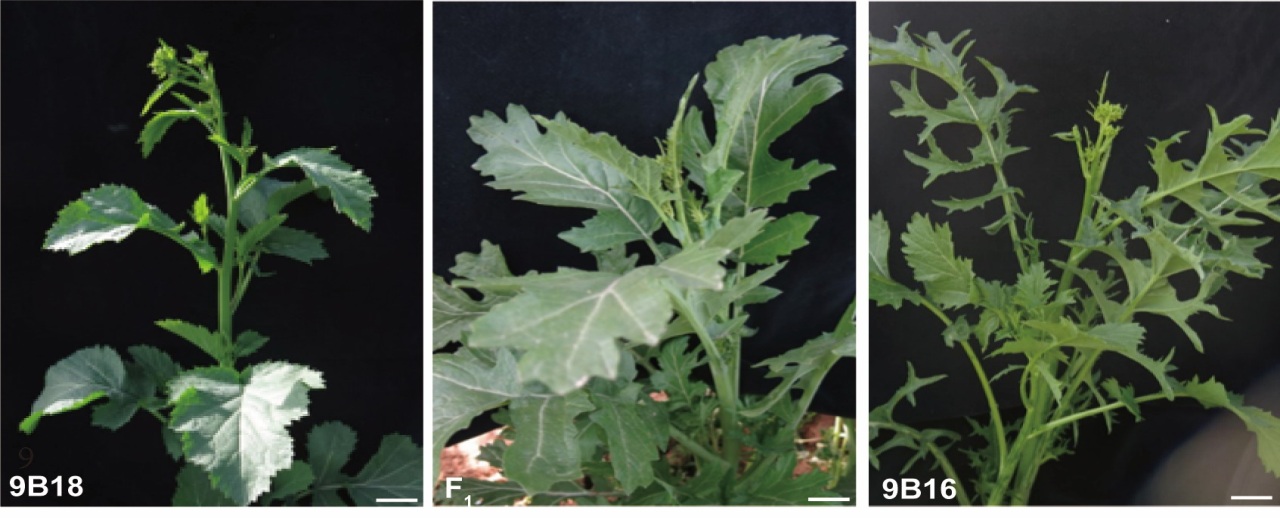


**Fig. S1** The characteristics of the leaf lobes of entire-leaf blades (9B18), lobed-leaf blades (9B16) and their F_1_ hybrid leaves.

9B16 has obviously deeply lobed leaves, while their F_1_ hybrid shows serrated leaves, and these serrated leaves are similar to those of the lobed-leaf parent 9B16. Scale bar = 2 cm.


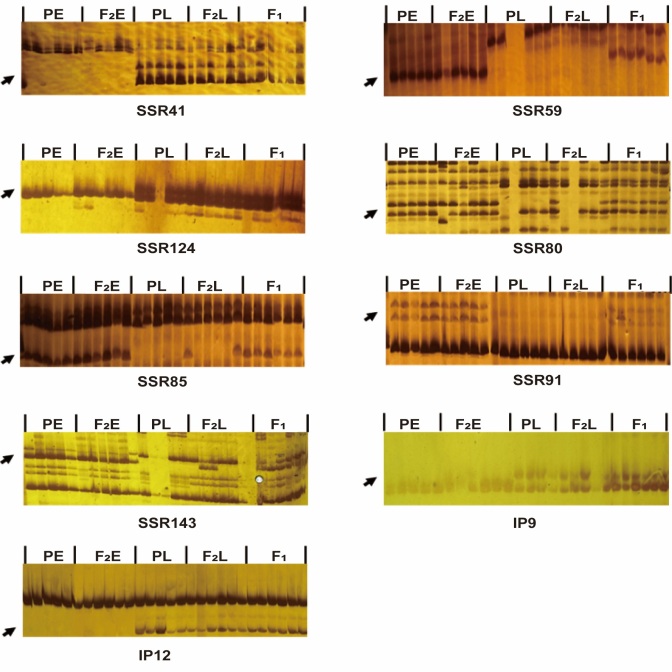


**Fig. S2** Verification results of molecular markers in the parental plants and individual plants with entire and lobed leaves in the F_2_ generation.

PE: DNA from 5 individual parental plants with entire leaves; PL: DNA from 5 individual parental plants with lobed leaves; F_1_: DNA from 6 individual plants of the F_1_ generation; F_2_E: DNA from 6 individual plants with entire leaves in the F_2_ generation; F_2_L: DNA from individual plants with lobed leaves in the F_2_ generation. Arrows indicate the target bands.


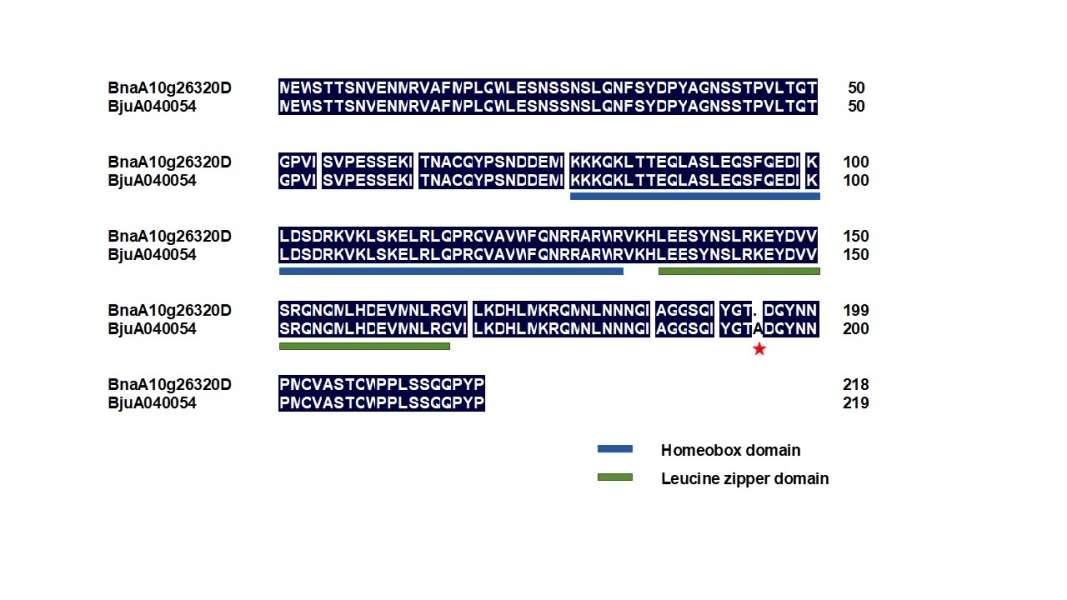


**Fig. S3** Alignment of amino acid sequences of candidate genes.


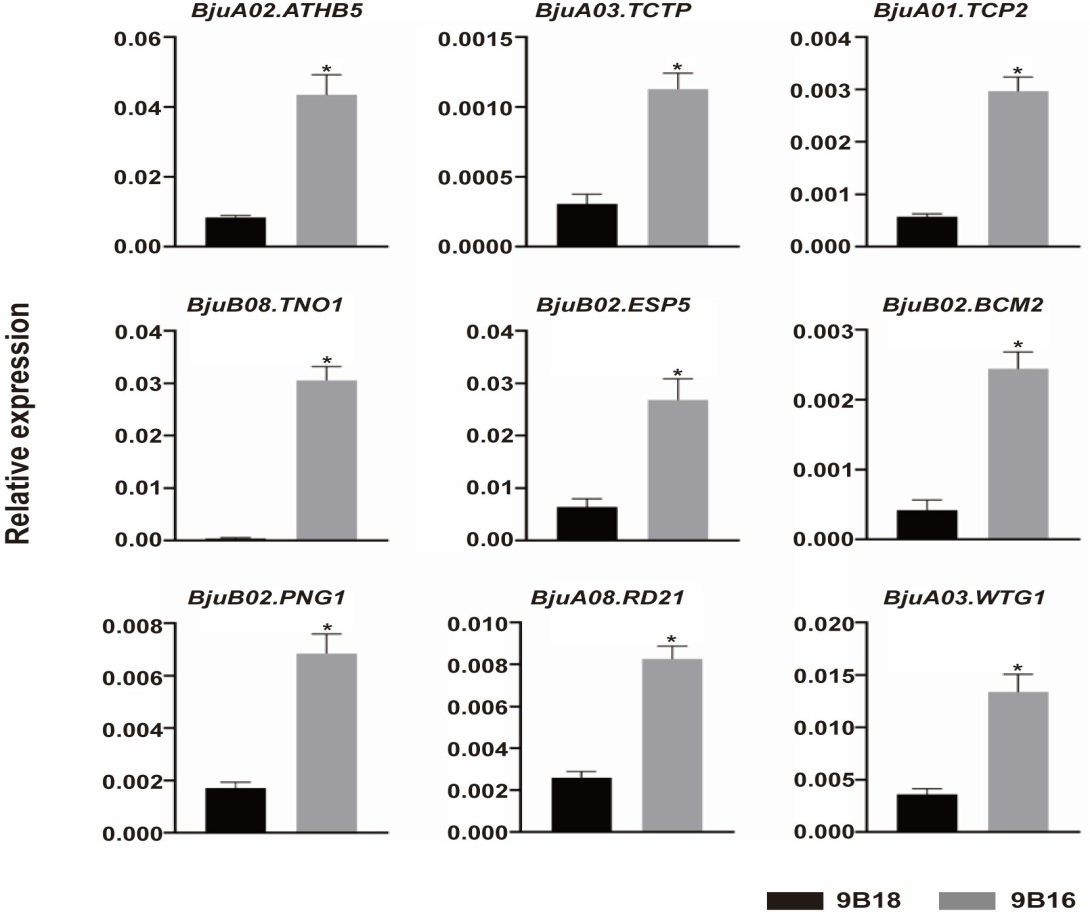


**Fig. S4** RT-qPCR validation of some differentially expressed genes in the transcriptome.

Results were normalized to *BjuUBQ9* as an internal reference gene. Values are presented as mean ± standard deviation (*n* = 3). (*) indicates significant difference between 9B16 and 9B18 (two-tailed paired Student’s *t*-test, *P* ≤ 0.05).


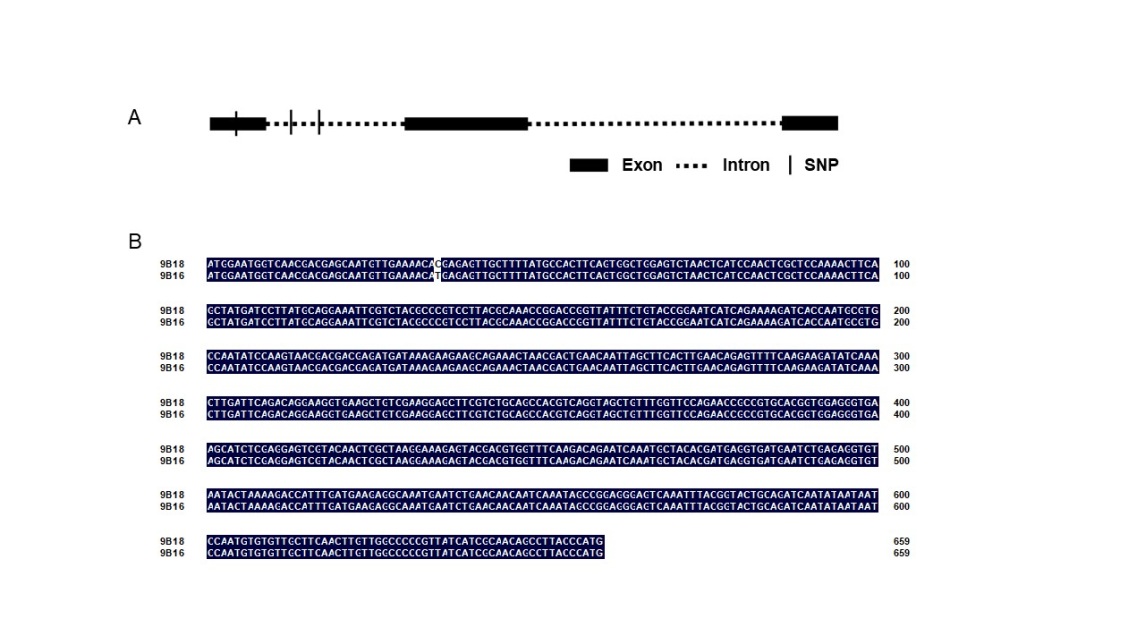


**Fig. S5** Sequence analysis of *BjA10.LL*.

(A) Gene structure of *BjA10.LL*. (B) Sequence alignments of the cDNA sequence of *BjA10.LL* in lines 9B18 and 9B16.


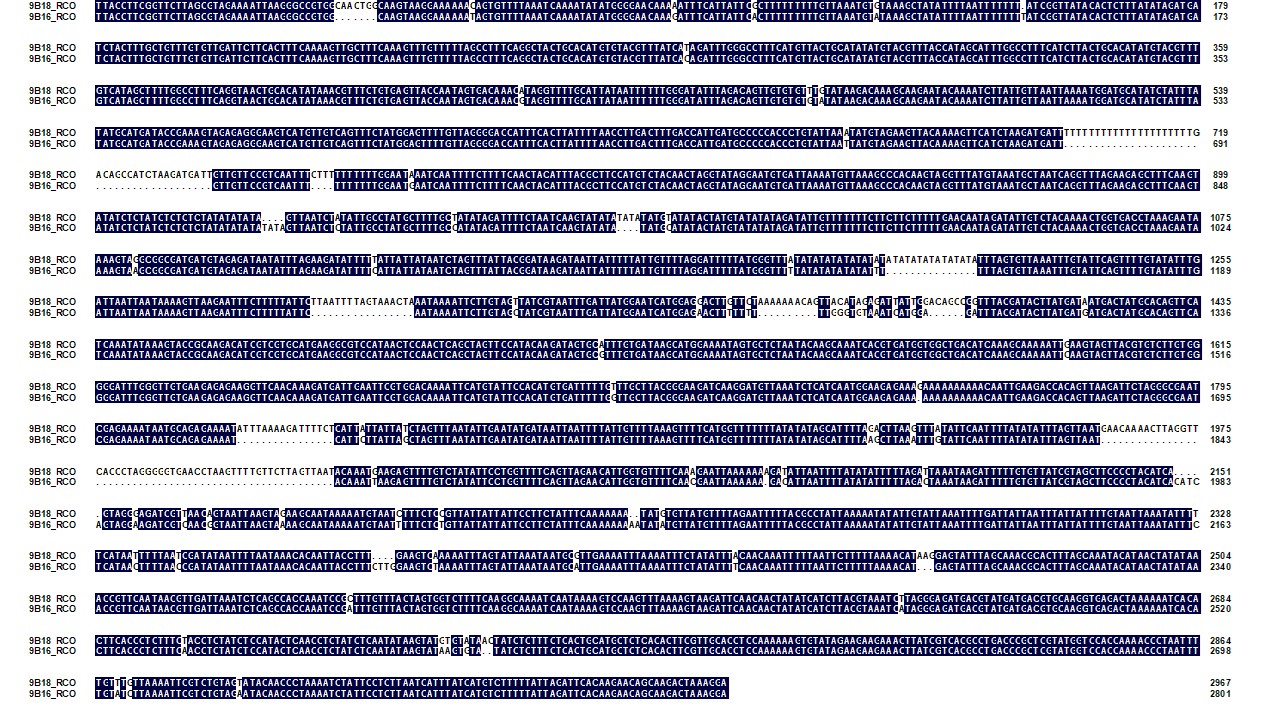


**Fig. S6** Sequence alignments of the ~ 3.0 kb region upstream of *BjA10.LL* start codon between 9B18 and 9B16.


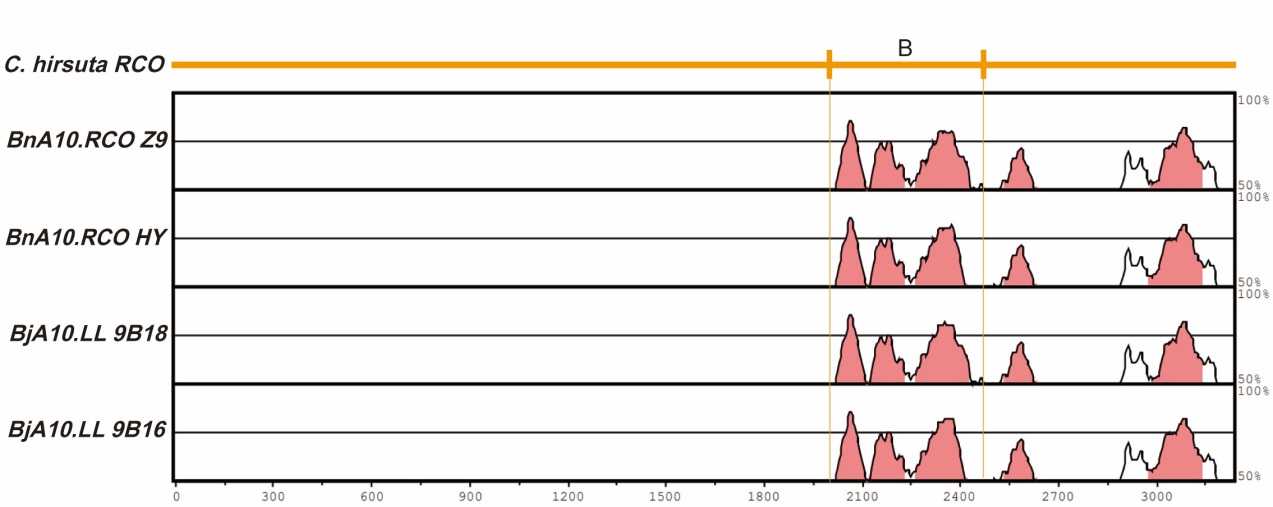


**Fig. S7** mVISTA graph of the alignment of the upstream sequence of the *BjA10.LL* gene in *B. juncea* with the *RCO* genes in *B. napus* and of *C. hirsuta*. Nucleotide positions along the X-axis are annotated relative to the transcriptional initiation site (start codon) of the target gene. The genomic interval designated as Region B corresponds to the enhancer regulatory element.
